# Surface Roughness Optimization of Polyamide-6/Nanoclay Nanocomposites Using Artificial Neural Network: Genetic Algorithm Approach

**DOI:** 10.1155/2014/485205

**Published:** 2014-01-21

**Authors:** Mehdi Moghri, Milos Madic, Mostafa Omidi, Masoud Farahnakian

**Affiliations:** ^1^Islamic Azad University of Kashan, Ghotbe Ravandi Boulevard, Kashan 87159 98151, Iran; ^2^Faculty of Mechanical Engineering, University of Niš, Niš, Serbia; ^3^Islamic Azad University, Badroud Branch, Badroud, Iran; ^4^Islamic Azad University, Najafabad Branch, Najafabad, Iran

## Abstract

During the past decade, polymer nanocomposites attracted considerable investment in research and development worldwide. One of the key factors that affect the quality of polymer nanocomposite products in machining is surface roughness. To obtain high quality products and reduce machining costs it is very important to determine the optimal machining conditions so as to achieve enhanced machining performance. The objective of this paper is to develop a predictive model using a combined design of experiments and artificial intelligence approach for optimization of surface roughness in milling of polyamide-6 (PA-6) nanocomposites. A surface roughness predictive model was developed in terms of milling parameters (spindle speed and feed rate) and nanoclay (NC) content using artificial neural network (ANN). As the present study deals with relatively small number of data obtained from full factorial design, application of genetic algorithm (GA) for ANN training is thought to be an appropriate approach for the purpose of developing accurate and robust ANN model. In the optimization phase, a GA is considered in conjunction with the explicit nonlinear function derived from the ANN to determine the optimal milling parameters for minimization of surface roughness for each PA-6 nanocomposite.

## 1. Introduction

The key change drivers in the case of cutting technology include diminishing component size, enhanced surface quality, tighter tolerances and manufacturing accuracies, reduced costs, diminished component weight, and reduced batch sizes [1]. The surface quality of the machined parts is one of the most important product quality characteristics and one of the most frequent customer requirements. Surface quality is often expressed by the measurement of surface roughness. The surface roughness greatly affects the functional performance of mechanical parts such as wear resistance, fatigue strength, ability of distributing and holding a lubricant, heat generation and transmission, and corrosion resistance [2].

During the past decade, polymer nanocomposites have emerged relatively as a new, novel, and rapidly developing class of composite materials and attracted considerable investment in research and development worldwide. Nanocomposites are a class of composite materials where one of the constituents has at least one dimension in the range between 1 and 100 nm. Recent and ongoing research on polymer/inorganic nanocomposites has shown remarkable improvement of properties compared with pure polymer and their conventional microcomposites, even at very low filler content. One particular class of nanocomposites with great potential is polymer/clay nanocomposites [3, 4].

Polymer nanocomposites based on polyamide-6 (PA-6) have generated much interest in recent years because of their readily tailored properties. PA-6 itself is one of the most widely used engineering polymers because of its stiffness and strength. The key to enhanced properties for a polymer-clay nanocomposite such as PA-6/clay is delamination and dispersion of nanoscale layered clay fillers throughout the polymer matrix. In general, PA-6 is able to easily intercalate into clay layers to form exfoliated clay structure due to strong interaction between PA-6 and clay generated by the polarity and hydrogen-bonding capacity [5].

The composite materials have particular characteristics, which drive their machining behavior; the mechanisms involved while cutting composite materials have been regarded as considerably distinct from those observed when cutting homogeneous materials [6]. Surface roughness of a nanocomposite material, as for other materials, is also contributed by tool geometry and material properties, cutting kinematics, and cutting conditions [7]. To reduce machining costs and to obtain required surface quality of the machined parts, it is necessary to exactly quantify the relationship between surface roughness and cutting conditions through mathematical modeling and subsequently determine optimal machining conditions by using an optimization technique.

The comprehensive review papers by Benardos and Vosniakos [8], Feng and Wang [9], Dhokia et al. [10], and Pontes et al. [11] showed that most of the previous studies have been focused on modeling surface roughness in machining of metallic materials. While there is explicit information available on the machining of metals, knowledge regarding the machining of polymers and their nanocomposites is limited [12].

In recent years, there are few works on machining of polymers and their nanocomposites in milling and turning operations. Davim and Mata [13] presented an experimental study on the cutting process for polyamides PA-6 and PA-66/GF30 (reinforced with 30% glass fiber) using cemented carbide (K15) tools in turning [13]. In another work, Davim et al. studied the machinability of PA-66 with and without 30% glass fiber reinforcing, when precision turning at different feed rates and using four distinct tool materials [14]. Gaitonde et al. [15] analyzed the effects of process parameters (work material, type of cutting tool, cutting speed, and feed rate) on machinability characteristics (machining force, power, and specific cutting force) during turning of unreinforced polyamide PA-6 and reinforced polyamide PA-66/GF30 through artificial neural network (ANN) modeling. Gaitonde et al. [16] applied Taguchi's quality loss function approach for simultaneous minimization of the cutting power and specific cutting force during turning of both PA-6 and PA-66/GF30 polyamides. Taguchi's optimization methodology was performed with tool material, feed rate, and cutting speed as the process parameters. In another work, Gaitonde et al. [17] developed second-order response surface mathematical models for analyzing the influence of cutting speed and feed rate on machining force, cutting power, and specific cutting pressure during turning of unreinforced polyamide PA-6 and reinforced polyamide PA-66/GF30 [17]. Dhokia et al. [10] investigated the machining of soft materials with a focus on defining the optimum machining parameters for slot milling of polypropylene. The authors developed predictive ANN surface roughness model in terms of spindle speed, feed rate, and the depth of cut as the process parameters. In another work, Dhokia et al. [18] developed a predictive model using the design of experiments method to obtain optimized machining parameters, by utilizing genetic algorithm (GA), for a specific surface roughness in ball-end machining of polypropylene. Farahnakian et al. [12] investigated the influence of the cutting parameters (spindle speed and feed rate) and nanoclay content (NC) on machinability characteristics in milling of PA-6/NC nanocomposites. Cutting force and surface roughness were separately modeled by using a particle swarm optimization-based ANN. Yilmaz et al. [19] developed an ANN model for predicting the surface roughness in machining of PA-6 cast polyamide. The model was developed in terms of spindle speed and feed rate. By simulating the trained ANN for many input combinations, the response surface of the surface roughness was obtained from which the optimal cutting parameters were identified. Madić et al. [20] applied ANN based mathematical modeling in order to relate the cutting parameters (cutting speed, feed rate, depth of cut, and tool nose radius) and surface roughness in turning of polyamide material. By applying the simplex optimization method, the optimal cutting parameter settings, minimizing surface roughness, were determined.

The mathematical models of surface roughness are very useful for the proper planning and control of the machining operation and can serve as objective functions for the optimization of the machining conditions aiming to reduce production costs and time. Therefore, this paper aims at mathematical modeling of the relationships among the machining parameters (spindle speed and feed rate) and NC content, and surface roughness in milling of PA-6/NC nanocomposites. As noted by Erkan et al. [21] instead of expensive and time-consuming experiments, it is highly recommended the usage of ANNs for modeling composite materials machining processes. Therefore, in this study, mathematical model for surface roughness is based on ANN. As the present study deals with relatively small number of data, training ANN using RCGA is thought to be an appropriate approach for the purpose of developing accurate and robust ANN model for prediction of the surface roughness. In addition to modeling, the surface roughness mathematical model was optimized. Using the integrated ANN-GA approach, optimal machining conditions for each PA-6/NC nanocomposite material were determined.

## 2. Experimental Procedure

### 2.1. Work Piece Material

The PA-6 used in this work was B5 from BASF. The nanofiller was Nanofil 9 provided by Southern Clay Products which is organically modified montmorillonite with good adhesion to PA-6. PA-6 pellets and NC powder remained at 90°C for 12 hours and were mixed with high speed mixer at dry conditions. Nanocomposites with 2 and 6 phr nanoclay were prepared by melt mixing using a lab scale corotating twin-screw extruder (ZSK25, L/D = 40). The screw configuration used includes two high mixing zones using kneading elements and enhances dispersive and distributive mixing in the system, and the extruder was equipped with a circular die. Then, dry extruded pellets were injection molded into standard (ASTM D 638) tensile bars using 3 tons Engel injection molding machine. After molding, the specimens were sealed and placed in a vacuum desiccator for a minimum of 24 h prior to mechanical testing, under dry conditions.

### 2.2. Machining Conditions

The milling experiment was carried out in nanocomposite plates with 3 mm of thickness, using a two flute high-speed-steel (HSS) end mill, with 3 mm diameter. All experimental trials were performed on a Deckel Maho DMU 70 V vertical axis computer numerical control milling machine with a maximum spindle speed of 3,150 rpm and 3,000 mm/min of maximum feed rate. A constant depth of cut of 1 mm was used using HSS end mill.

### 2.3. Cut Quality Evaluation

In the study, the average surface roughness (*R*_*a*_) was used to evaluate the surface cut quality. Mathematically, *R*_*a*_ is the arithmetic value of the departure of profile from the centerline along sampling length. The average surface roughness of machined work pieces was measured using a Hommel Tester T8000 device. Three small regions on the machined surface were determined for measurements. Measurements in these regions were conducted and the average value was recorded as the *R*_*a*_. The tracing velocity and the cut-off lengths were fixed at 0.5 mm/s and 2.5 mm, respectively [12].

### 2.4. Experimental Plan

To develop mathematical model based on ANN that relates the milling parameters (spindle speed and feed rate) and NC content and average surface roughness (*R*_*a*_), a 3^3^ full factorial design was employed. The milling parameter values and NC content values considered are given in Table 1.

## 3. Mathematical Modeling of Surface Roughness

Surface roughness greatly affects the functional performance of mechanical parts such as wear resistance, fatigue strength, thermal conductivity, ability of distributing and holding a lubricant, heat generation and transmission, and corrosion resistance. Hence, it is of great importance to exactly quantify the relationship between surface roughness and cutting parameters so as to predict its value for any machining condition. In the present paper, for developing the mathematical relation between input parameters (NC content, spindle speed, and feed rate) and average surface roughness (*R*_*a*_), feed-forward ANN was used. The development, training, and testing of the ANN model were performed in MATLAB software package.

### 3.1. ANN Surface Roughness Model

#### 3.1.1. ANN Design

Artificial neural networks (ANNs) are massive parallel systems made up of numerous simple processing units called neurons that are linked with weighted connections. ANNs are characterized by their architecture, weight vectors and biases, and activation functions that are used in hidden and output layers [20]. ANN architecture, that is, the number of hidden layers and corresponding hidden neurons, and type of activation functions are to be specified prior to ANN training, by which weight vectors and biases, which are initially assigned to small random numbers, are determined using a training algorithm. ANN training is considered as one of the most important steps in ANN model development [21]. The primary goal of ANN training is to achieve a good balance between the ANN ability to respond correctly to the input data used for the training and, more preferably, the ability to produce accurate predictions to input that is not used in training (generalization ability).

Three neurons in the input layer for representing the NC content, spindle speed, and feed rate, one neuron in the output layer for calculating (predicting) the surface roughness, and only one hidden layer were used to define ANN architecture. This ANN was chosen, because it is widely reported that single hidden layer ANN can be trained to approximate most functions arbitrarily well. Since it was assumed that there exists nonlinear relationship between input and output parameters, the hyperbolic tangent sigmoid transfer function was used in the hidden layer, and linear transfer function was used in the output layer. In order to stabilize and enhance ANN training the input and output data were normalized in [−1,1] range.

#### 3.1.2. ANN Training

The most common training algorithm for ANNs is the back-propagation (BP) algorithm and its variants, because it is stable and easy to implement. However, the convergence speed of the BP gradient descent approach can be significantly affected by initial weights settings and values of the algorithm parameters which are usually set by previous experience in trial and error procedure. Furthermore, because ANNs generate complex error surfaces with multiple local optima, even for simple functions being estimated, the BP tends to become trapped in local solutions that are not global [22]. Finally, the basic BP algorithm is often very slow to converge in real practice. Considering the disadvantages of the BP, in recent years metaheuristic algorithms, particularly GAs, have been applied to ANN training. These techniques have been shown to be very effective in solving hard optimization problems like training of ANNs, that is, problems in which gradient-descent techniques get trapped into local minima, or are fooled by the complexity and/or nondifferentiability of the search space [23]. As noted by Blanco et al. [24], real coding is the most suitable coding for continuous domains and it is beneficial to use real-coded genetic algorithm (RCGA) for the ANN training purpose. As the present study deals with relatively small number of data obtained from full factorial experimental design, ANN training using RCGA is thought to be an appropriate approach for ANN training.

Out of the 27 experimental data, 24 data were considered for ANN training and the rest were used for ANN testing. The RCGA itself is not discussed and the details are available elsewhere along with numerous examples of applications [24–26]. The aim of the RCGA was to explore search space to find near optimal weights and biases on the ANN. These include weights between the input layer and the hidden layer, weights between the hidden layer and the output layer, biases of the hidden neurons and bias of the output neuron. The objective of the RCGA application was to approximate weights and biases such that to minimize the following fitness function:

(1) $$\begin{array}{c} E=\sum_{i=1}^{24}\left|y_{i}-{\hat{y}}_{i}\right|, \end{array}$$

where *y*_*i*_ and ${\hat{y}}_{i}$ represent experimental and ANN predicted values of surface roughness for the training sample *i*, respectively.

**Figure 1:**
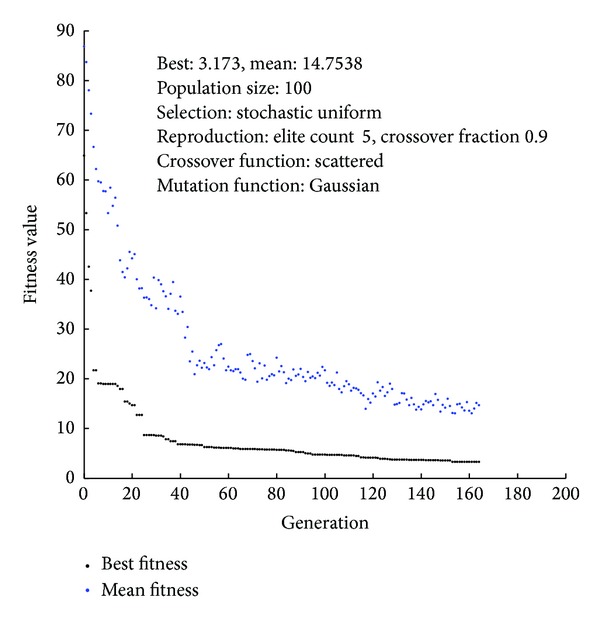
Plot functions of the best fitness.

Basically, obtaining the best optimal results depends on some features related with the RCGA parameters. Although some general guidelines about such selections exist in relevant literature, it was reported that optimal settings are strongly related to the design problem under consideration. Here it should be noted that a different number of hidden neurons were tried. The number of hidden neurons was altered by considering that too few neurons can lead to underfitting, whereas too many neurons can contribute to overfitting, and that more expressive power of the ANN comes with more hidden neurons. Also, the well-known bias-variance trade-off in ANN model development was considered [27]. After a number of trials were conducted with different parameter settings of the RCGA it was found that 3-5-1 ANN architecture provided best data fitting capability, after tradeoff.  Figure 1 shows convergence of the optimization problem and the RCGA parameter values used for ANN training.

After an iterative calculus, RCGA provided the (near) optimal values for weights between the input layer and the hidden layer (*w*_*ji*_), weights between the hidden layer and the output layer (*w*_*kj*_), biases of the hidden neurons (*b*_*j*_), and bias of the output neuron (*b*_*k*_). The weights and the biases of the RCGA trained ANN surface roughness model are given in Table 2.

Regarding the used activation functions in hidden and output layers, and by using the weights and biases from Table 2, one can develop exact mathematical relationship between the average surface roughness (*R*_*a*_) and input parameters so as it is possible to calculate *R*_*a*_ value for the given machining conditions. After denormalization, the mathematical model for the *R*_*a*_ in terms of the input parameters can be expressed by the following equation:

(2) $$\begin{array}{c} R_{a}=2.55\cdot \left(\left[\frac{2}{1+e^{-2(X\cdot w_{ji}+b_{j})}}-1\right]\cdot w_{kj}+b_{k}+1\right)+2.1, \end{array}$$

where *X* is the column vector which contains normalized values of input parameters.

#### 3.1.3. ANN Model Validation

**Figure 2:**
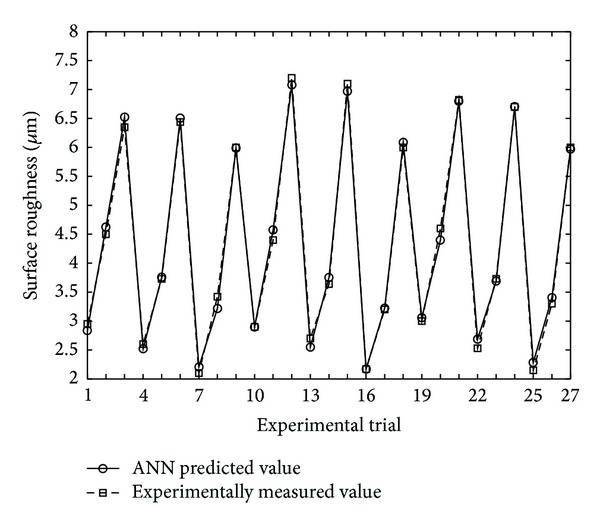
Comparison of experimentally measured and ANN predicted values.

In order to check the reliability of the developed ANN model, the prediction accuracy was tested. Using (2) the predicted and experimentally measured surface roughness values are compared in Figure 2.

In addition, the absolute percentage errors were calculated for training and testing data independently. The mean absolute percentage errors using training and testing data were found to be 2.49% and 2.99%, respectively. These results indicate that the developed mathematical model has good accuracy for predicting the surface roughness within the scope of machining conditions investigated in the study and hence can be used for the analysis and optimization of the surface roughness.

## 4. Optimization of Surface Roughness

### 4.1. Optimization Problem Formulation

An appropriate selection of machining parameter values would increase the product quality by minimizing the average surface roughness (*R*_*a*_) as the main indicator of surface quality. In order to get the optimal machining parameters, that is, the combination of the spindle speed and feed rate that gives the minimal value of the *R*_*a*_, the optimization using RCGA was conducted. The goal of the optimization process in this study is to determine the optimal spindle speed and feed rate values that contribute to the minimum value of the *R*_*a*_ for pure PA-6, PA-6 with 2% NC, and PA-6 with 6% NC. The milling optimization problem was defined as below:

(3a) Find: f,nto minimize: Ra=f(NC content,f,n)

(3b) subject to:  0.03≤f≤0.11 (mm/tooth)  40≤v≤630 (rpm)  NC content={0,2,6}.

### 4.2. Surface Roughness Optimization by RCGA

For calculating average surface roughness (*R*_*a*_) in previous equation, the mathematical function based on the developed ANN (2) was used. The optimization solution results were obtained using the default parameter settings of the RCGA provided by MATLAB optimization toolbox. The optimization results are summarized in Table 3.

**Figure 3:**
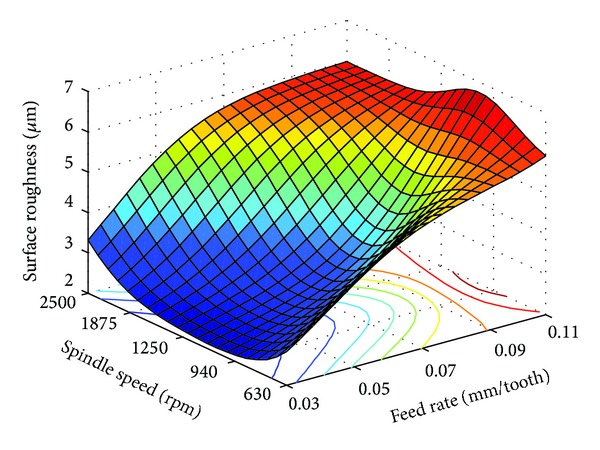
Surface plot of interaction effects of milling parameters on the surface roughness for pure PA-6.

**Figure 4:**
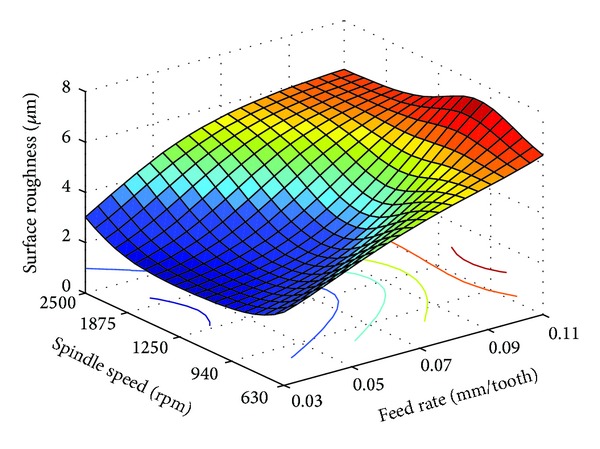
Surface plot of interaction effects of milling parameters on the surface roughness for PA-6 with 2% nanoclay.

**Figure 5:**
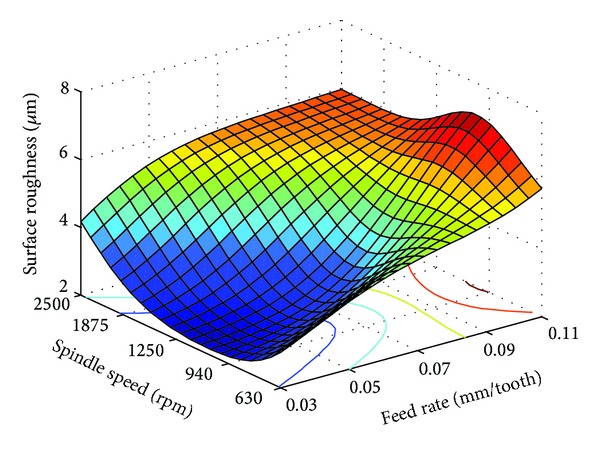
Surface plot of interaction effects of milling parameters on the surface roughness for PA-6 with 6% nanoclay.

From Table 3, it is seen that for all three materials feed rate converged to the lower limits, while spindle speed converged to intermediate levels. This indicates that RCGA optimized surface roughness is directly proportional with feed rate, and in the case of spindle speed, optimum surface roughness can be achieved when the spindle speed is set nearer to the intermediate levels. Using (2), Figures 3, 4, and 5 were generated from the functional dependence between the surface roughness and interaction of the spindle speed and feed rate for PA-6 and different nanocomposites samples.

As can be seen from Figures 3 to 5, the RCGA optimization results of Table 3 can be confirmed. Actually, as can be seen in these figures, for each of the three different samples considered, there exists a region of optimal combinations of feed rate and spindle speed which produces low surface roughness. In terms of milling parameter values and corresponding average surface roughness values these regions are very similar. From the analysis of these figures, it can be concluded that nanoclay content does not have considerable influence on surface roughness. This may be related to the low shear stress of the PA-6 and its nanocomposites [12].

As shown in Figures 3 to 5, both effects of the feed rate and spindle speed on the surface roughness are variable, nonlinear, and interdependent. Generally, an increase in the feed rate increases surface roughness. For the feed rate considered in this study, the effect of the spindle speed on the surface roughness is variable. For feed rates below 0.07 mm/tooth, in the first stage the surface roughness decreases with the increase of spindle speed, but after a certain limit, the increase of spindle speed increases the surface roughness, and this is valid for PA-6 sample and PA-6/NC nanocomposites samples. At higher feed rate of 0.07 to 0.11 mm/tooth, the increase in spindle speed from 1250 to 2500 rpm has no significant effect on the surface roughness. For the same feed rate range, change in the spindle speed bellow 1250 rpm adversely affects surface roughness. It can be also observed that the interaction effect between feed rate and spindle speed is more pronounced at higher and lower feed rates. From the analysis of Figures 3 to 5 it can be also concluded that both milling parameters have significant effect on the surface roughness for all of the three samples; however, the effect of the feed rate is more pronounced.

In some cases, for technical-technological and/or other reasons, it is not possible to use the optimal values of machining parameters, for the chosen optimization criteria. However, on the basis of the generated plots as shown in Figures 3 to 5, one can select a number of milling parameter combination values so as to achieve desired surface finish.

## 5. Conclusions

This study presented an integrated ANN-GA approach for modeling and optimization of surface roughness in milling PA-6/NC nanocomposites. The statistical results indicate that the proposed approach can be used efficiently for accurate modeling of relationships in milling of PA-6/NC nanocomposites. On the basis of the experimental results and derived analysis, the surface roughness is highly sensitive to the selected milling parameters and their interactions. The functional dependence between the surface roughness and milling parameters is non-linear and variable. Surface roughness increases when the feed rate increases, and this is valid for PA-6 and different nanocomposites samples. Generally an increase in the spindle speed increases the surface roughness; however, the effect of spindle speed on the surface roughness is variable and must be considered through the interaction with the feed rate. From the derived analysis it was observed that, for all of the considered samples, there exists an optimal spindle speed which yields minimal surface roughness. The feed rate has the maximum influence on the surface roughness followed by the spindle speed, whereas the addition of nanoclay to the PA-6 has negligible influence on the surface roughness. Based on optimization results, it was observed that surface roughness in milling of PA-6/NC nanocomposites is minimal at the lowest level of feed rate and an intermediate level of spindle speed.

## Figures and Tables

**Table 1:** Independent parameters and their levels.

Independent variables	Level 1	Level 2	Level 3
NC content (phr)	0	2	6
Spindle speed (rpm)	630	1250	2500
Feed rate (mm/tooth)	0.03	0.07	0.11

**Table 2:** Weights and biases after ANN training.

*w*_*ji*_			*w*_*kj*_	*b*_*j*_	*b*_*k*_
1.609	0.21574	1.6796	0.081196	−2.3971	0.4018
−0.19979	−1.5177	−1.8131	−0.67489	0.4699	—
1.9631	−1.0908	−0.24814	−0.22426	0.69287	—
2.5242	0.023746	−0.87057	0.13252	0.97903	—
0.084185	1.29	−2.4474	0.56262	−1.5756	—

**Table 3:** RCGA optimization results.

Material	Surface roughness (μm)	Feed rate (mm/tooth)	Spindle speed (rpm)
Pure PA-6	2.1272	0.03	1326.17
PA-6 with 2% nanoclay	1.948	0.03	1642.1
PA-6 with 6% nanoclay	2.176	0.03	1318.8

## References

[1] Byrne G, Dornfeld D, Denkena B (2003). Advancing cutting technology. *CIRP Annals*.

[2] Velibor M, Miloš M (2011). Optimization of surface roughness in turning alloy steel by using taguchi method. *Scientific Research and Essays*.

[3] Alexandre M, Dubois P (2000). Polymer-layered silicate nanocomposites: preparation, properties and uses of a new class of materials. *Materials Science and Engineering R*.

[4] Osman MA, Mittal V, Lusti HR (2004). The aspect ratio and gas permeation in polymer-layered silicate nanocomposites. *Macromolecular Rapid Communications*.

[5] Moghri M, Garmabi H (2008). Investigation of the effects of formulation and processing parameters on properties of PA 6 nanocomposites using Taguchi method of experimental design. *International Polymer Processing*.

[6] Davim JP, Reis P (2003). Drilling carbon fiber reinforced plastics manufactured by autoclave-experimental and statistical study. *Materials and Design*.

[7] Dagnall H (1986). *Exploring Surface Texture*.

[8] Benardos PG, Vosniakos G-C (2003). Predicting surface roughness in machining: a review. *International Journal of Machine Tools and Manufacture*.

[9] Feng C-X, Wang X-F (2003). Surface roughness predictive modeling: neural networks versus regression. *IIE Transactions*.

[10] Dhokia VG, Kumar S, Vichare P, Newman ST, Allen RD (2008). Surface roughness prediction model for CNC machining of polypropylene. *Proceedings of the Institution of Mechanical Engineers B*.

[11] Pontes FJ, Ferreira JR, Silva MB, Paiva AP, Balestrassi PP (2010). Artificial neural networks for machining processes surface roughness modeling. *International Journal of Advanced Manufacturing Technology*.

[12] Farahnakian M, Razfar MR, Moghri M, Asadnia M (2011). The selection of milling parameters by the PSO-based neural network modeling method. *International Journal of Advanced Manufacturing Technology*.

[13] Davim JP, Mata F (2007). A comparative evaluation of the turning of reinforced and unreinforced polyamide. *International Journal of Advanced Manufacturing Technology*.

[14] P. Davim J, Silva LR, Festas A, Abrão AM (2009). Machinability study on precision turning of PA66 polyamide with and without glass fiber reinforcing. *Materials and Design*.

[15] Gaitonde VN, Karnik SR, Mata F, Davim JP (2010). Modeling and analysis of machinability characteristics in PA6 and PA66 GF30 polyamides through artificial neural network. *Journal of Thermoplastic Composite Materials*.

[16] Gaitonde VN, Karnik SR, Mata F, Davim JP (2008). Taguchi approach for achieving better machinability in unreinforced and reinforced polyamides. *Journal of Reinforced Plastics and Composites*.

[17] Gaitonde VN, Karnik SR, Mata F, Davim JP (2009). Study on some aspects of machinability in unreinforced and reinforced polyamides. *Journal of Composite Materials*.

[18] Dhokia VG, Kumar S, Vichare P, Newman ST (2008). An intelligent approach for the prediction of surface roughness in ball-end machining of polypropylene. *Robotics and Computer-Integrated Manufacturing*.

[19] Yilmaz S, Arici AA, Feyzullahoglu E (2011). Surface roughness prediction in machining of cast polyamide using neural network. *Neural Computing and Applications*.

[20] Madić M, Marinković V, Radovanović M (2012). Mathematical modeling and optimization of surface roughness in turning of polyamide based on artificial neural network. *Mechanics*.

[21] Benardos PG, Vosniakos GC (2002). Prediction of surface roughness in CNC face milling using neural networks and Taguchi's design of experiments. *Robotics and Computer-Integrated Manufacturing*.

[22] Gupta JND, Sexton RS (1999). Comparing backpropagation with a genetic algorithm for neural network training. *Omega*.

[23] Alba E, Martí R (2006). *Metaheuristic Procedures for Training Neural Networks*.

[24] Blanco A, Delgado M, Pegalajar MC (2001). A real-coded genetic algorithm for training recurrent neural networks. *Neural Networks*.

[25] Kim SS, Kim I-H, Mani V, Kim HJ (2008). Real-coded genetic algorithm for machining condition optimization. *International Journal of Advanced Manufacturing Technology*.

[26] Shen C, Wang L, Li Q (2007). Optimization of injection molding process parameters using combination of artificial neural network and genetic algorithm method. *Journal of Materials Processing Technology*.

[27] Feng C-XJ, Yu Z-G, Kusiak A (2006). Selection and validation of predictive regression and neural network models based on designed experiments. *IIE Transactions*.

